# Virtual team-cooperation from home-office: a quantitative diary study of the impact of daily transformational- and passive-avoidant leadership – and the moderating role of task interdependence

**DOI:** 10.3389/fpsyg.2023.1188753

**Published:** 2023-06-02

**Authors:** Olav Kjellevold Olsen, Kari Wik Ågotnes, Jørn Hetland, Roar Espevik, Conrad Alexander Ravnanger

**Affiliations:** ^1^Department of Psychosocial Sciences, University of Bergen, Bergen, Norway; ^2^Department of Leadership and Organizational Behavior, BI Norwegian Business School, Oslo, Norway; ^3^VID Specialized University, Oslo, Norway

**Keywords:** virtual team cooperation, transformational leadership, passive-avoidant leadership, task interdependence, homeoffice, COVID-19

## Abstract

During the Covid-19 pandemic, most of the workforce moved from office setting to home-office and virtual teamwork. Whereas the relationship between leadership and team cooperation in physical settings is well documented – less is known about how daily virtual team cooperation is influenced by daily constructive as well as destructive leadership, and how intervening mechanisms influence this relationship. In the present study, we test the direct effect of daily transformational- and passive avoidant leadership, respectively, on the daily quality of virtual team cooperation – and the moderating effect of task interdependence. Using virtual team cooperation as outcome, we hypothesized that (a) transformational leadership relates positively to virtual team cooperation, (b) passive-avoidant leadership relates negatively, and (c) moderated by task interdependence. Our hypotheses were tested in a 5-day quantitative diary study with 58 convenience sampled employees working from home in virtual teams. The results show that virtual team cooperation is a partially malleable process – with 28% variation in daily virtual team cooperation resulting from within team variation from day to day. Surprisingly, the results of multilevel modeling lend support only to the first hypothesis (a). Taken together, our findings suggest that in virtual settings, inspirational and development-oriented transformational leadership plays a key role in daily team cooperation, while passive-avoidance has little impact – independently of task interdependence. Hence, in virtual team settings, the study shows that “good is stronger than bad” – when comparing the negative effects of destructive leadership to the positive effect of constructive and inspirational leadership. We discuss the implications of these findings for further research and practice.

## Introduction

The 12th of March 2020, Norway suffered their first Covid-19 related death, and instant and extensive preventive measures from the Government was introduced, such as overnight deployment of virtual teams for most of the workforce. Today, virtual- and hybrid work arrangements can be seen as “the new normal” in many organizations around the world (e.g., Garro-Abarca et al., 2020; Brown et al., 2021; Marino-Romero et al., 2022). However, from a practitioner perspective, the experiences from virtual home-office appear mixed. On one side, positive outcome like work engagement and increased task performance are emphasized, while others experience fatigue and a sense of isolation and breaches of cohesion (e.g., Mortensen and Haas, 2021). The research literature also presents mixed results. For example, Anderson et al. (2015) found more job-related positive well-being and less negative on days when they were working from home, while Kurkland and Bailey (1999) reported negative outcomes like loneliness, lack of cooperation and impaired social networks.

In the literature, leadership is hypothesized as an important predictor of virtual team cooperation and performance (e.g., Brown et al., 2021; Garro-Abarca et al., 2021). However, what constitutes well-functioning virtual leadership appears ambiguous. Some claim that a virtual leader should be in the background, emphasizing shared or self-leadership practices (Lee-Kelley and Sankey, 2008; Angelo and McCarthy, 2020), while others promote high visibility and assertiveness (Bradley and Vozikis, 2004). Still, empirical evidence of the relationship between leadership and virtual team performance is relatively sparse (Bell et al., 2019; Kilcullen et al., 2022). Following Brown et al. (2021), both relationship- and task-oriented leadership may have a positive impact on virtual team cooperation, but more specific studies of the effects of transformational- as well as transactional leadership have produced mixed results (Bell et al., 2019). However, recent studies find transformational leadership to be a particularly effective strategy (Mysirlaki and Paraskeva, 2020; Sedrine et al., 2021). These studies, however, are limited by their cross-sectional research design – impairing the investigation of causality, mediations, and time-effects (Ohly et al., 2010). The notion of intra-individual stability embedded in this design may also cause important aspects of leadership and team behavior to be concealed. Thus, an increasing number of quantitative diary studies show that emotions, cognitions, and behaviors fluctuate from day-to-day, unrelated to more stable traits (Ohly et al., 2010). To circumvent this limitation, the present study utilizes a quantitative diary research design, proposing that variation in team cooperation will be partially explained by day-by-day changes within each team member. In this way, we can identify, not the best leaders (between-subjects analysis), but the best leadership, by studying the effects on the same individuals from day to day.

It is further noteworthy, according to recent literature reviews, that few, if any, have investigated how destructive leadership, and specifically passive-avoidant leadership influence virtual team cooperation (Bell et al., 2019; Brown et al., 2021). It could be expected that virtual leaders due to physical distancing, reduced availability, and fewer contact-points with followers, may overlook important challenges in the work group, for example related to conflicts, reduced motivation, and fatigue (Brown et al., 2021; Mortensen and Haas, 2021). This may be perceived by followers as passive and avoidant leader behavior, with subsequent impairment in performance and team cooperation consequently (e.g., Skogstad et al., 2014).

Finally, the mixed findings on virtual leadership and virtual work points to the relevance of including moderators in the research models (Bell et al., 2019). Few, if any, have investigated how task interdependence may influence the impact of virtual transformational and passive-avoidant leadership on team cooperation (e.g., Arnold and Tafkov, 2019). It could be expected that teams in which members are mutually dependent on each other to be able to do their jobs, will have greater need for leadership and will be more influenced by their leader. Conversely, teams with more independent members are likely to require less coordination by the leader.

**Figure 1:**
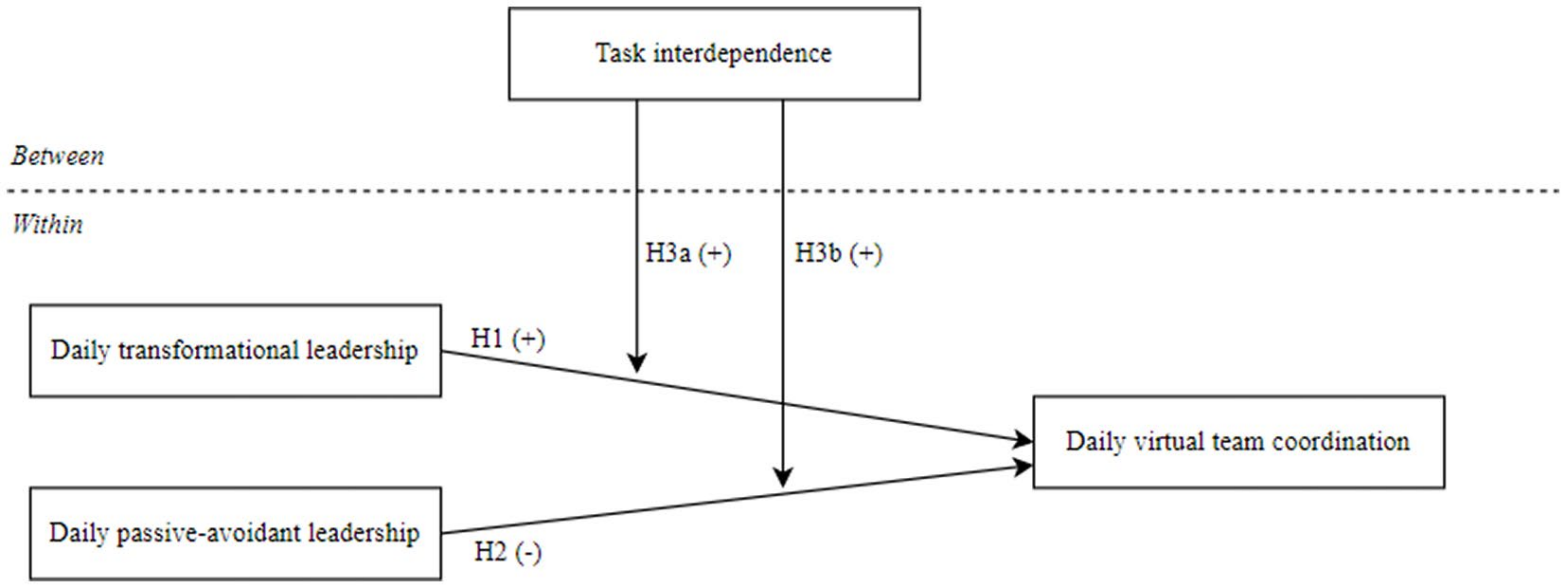
Research model.

At this backdrop, by using a quantitative diary study design, as illustrated in Figure 1, the present study investigates how (1) daily team cooperation is influenced by daily exposure to a) constructive transformational leadership and b) destructive passive-avoidant leadership behavior, respectively. Finally, (2) we test whether team characteristics in terms of task interdependence moderates this link between daily leadership and virtual team cooperation.

### Virtual team cooperation

According to Hertel et al. (2005, p. 71) “virtual teams consist of (a) two or more persons who (b) collaborate interactively to achieve common goals, while (c) at least one of the team members works at a different location, organization, or at a different time so that (d) communication and coordination is predominantly based on electronic communication media.” Several benefits have been attributed to virtual teamwork, including more diverse composition and expertise compared to conventional teams (Ebrahim et al., 2009), and more agile adoption to new demands by swift inclusion of experts beyond geographical boundaries. However, more complex coordination between team-members, difficulty in monitoring and controlling the work processes, and impaired communication have been identified as obstacles in virtual team cooperation (Crisp and Jarvenpaa, 2013; Dulebohn and Hoch, 2017; Van der Lippe and Lippényi, 2020).

In general, team cooperation can be seen as a dynamic sequence of behaviors and interactions between team members – encompassing monitoring, coordination, and communication (Healey et al., 2004). More specifically, Song et al. (2019) operationalize effective team cooperation, much in line with Salas et al. (2005) as a combination of (a) effective communication (b) agile coordination (c) mutual help and assistance (d) shared team goals (e) mutual work norms, and (f) cohesion and conflict resolution.

As noted, individual team members contribution to such team behaviors, and thus the team cooperation, may fluctuate from day to day, due to changes in emotions, cognitions, and behaviors that are unrelated to stable dispositions (Ohly et al., 2010). Given this dynamic, it is worth noting that several studies find that leadership may have significant impact on within-person variation on team relevant behaviors (e.g., Bakker et al., 2022). For example, Breevaart et al. (2014) found team members to be more engaged in their job on days with more exposure to transformational leadership.

### The impact of daily transformational leadership on daily virtual team cooperation

Effective leadership has long been seen as the outcome of behavior well-tailored to situational demands and resources (Bass and Riggio, 2006). In a virtual setting, the situational demands may be particularly related to physical distance, fewer informal meeting points, less physical interaction between team-members and difficulties in monitoring and helping each other during the workday (Garro-Abarca et al., 2020; Brown et al., 2021). This may, in turn, lead to increased misunderstandings, impaired coordination, and problems like reduced work engagement and withdrawal from work processes (Ziek and Smulowitz, 2014; Liao, 2017).

To tackle this, transformational leadership may be a particularly useful strategy – to address team needs in virtual settings. Transformational leadership denotes the process of a leader motivating followers to strive for group versus personal goals through charisma, inspirational motivation, intellectual stimulation, and individualized consideration (Bass, 1985). This represents influence-strategies with long range impact, transcending physical contact and interaction.

More specifically, idealized influence refers to followers’ admiration for their leader, who provides a clear vision (Bass and Riggio, 2006). This entails the ability to communicate symbolically and create a sense of hope that the vision will help the organization to thrive. Inspirational motivation encompasses the ability to inspire and motivate followers to reach ambitious goals by displaying optimism and enthusiasm that inspire followers to feel confident. Through intellectual stimulation, leaders challenge team members assumptions and solicit ideas from followers without criticism, which in turn may stimulate better decision making and how team members frame and think about obstacles and problems. Finally, individual consideration refers to leaders’ support and coaching, frequency of interaction, and attention to their followers to help them develop and grow. This requires an eye for individual needs and an awareness that each follower is unique. Taken together, transformational leadership involves a range of leader behaviors that may reduce the risk of social disintegration, reduced work engagement, and impaired interaction in virtual teams.

In a virtual setting, we suggest that communication of an inspiring vision and goals, in line with idealized influence, will create a common outlook and a strengthened team orientation among the team members, which may stimulate their understanding of, and attention to, the overall performance of the virtual team and the other team members’ work (Bass, 1985; Salas et al., 2005). Hence, a feeling of participating to something that is important, and something that must be tackled as a team, may increase mutual monitoring, task-related communication, and helping-behavior between the team members, which in turn may increase coordination and agility (Salas et al., 2005). Furthermore, a shared commitment may also increase the willingness and ability to manage team-conflicts among the team members, due to a shared fear that conflicts may jeopardize the mission.

Further, when a virtual leader engages in transformational behaviors, s/he acknowledges the unique knowledge, abilities, and skills of team members (i.e., individual consideration). This involves following up each of the team members on a personal level, through virtual channels, which may reduce the risk of team members feeling isolated and subsequently demotivated at their home office (Hetland et al., 2011). In the same vein, a 34-day diary study of military cadets found that exposure to daily transformational leadership increased a sense of social support in followers, which subsequently elevated their work engagement (Breevaart et al., 2014). In a virtual setting, this may be particularly relevant, due to the strain and stress that such settings may represent (e.g., Bell et al., 2019; Brown et al., 2021; Mortensen and Haas, 2021). By increasing social support, the members ability to bounce back, and maintain high performance and contribute to the team may be strengthened.

Finally, a recent diary study by Bakker et al. (2022) showed that daily transformational leadership, such as intellectual stimulation and individual consideration, stimulated followers’ use of personal strengths and personal initiative, which in turn boosted their work engagement. In a virtual home office setting, such personal initiative may be particularly important, increasing behavior like offering or asking for help, providing suggestions related to how the team could cooperate better, or discussing conflict-related issues that may disrupt a positive work climate.

At this basis, we hypothesize:

*Hypothesis 1*: Daily transformational leadership relates positively to the quality of daily team cooperation.

### The impact of daily passive-avoidant leadership on daily virtual team cooperation

Passive-avoidant leadership can be seen as a combination of management-by-exception passive and *laissez-faire* leadership (Skogstad et al., 2017). Management-by-exception passive can be seen as a reactive reaction from the leader to followers’ errors – after mistakes have occurred (Avolio and Bass, 2004), and *laissez-faire* leadership as “the absence of leadership, the avoidance of intervention, or both” (Bass and Avolio, 1994, p.20). Here, delayed decision making and no attempts to motivate team members or to recognize and satisfy their needs is a typical pattern, along with the absence of feedback, rewards, and involvement. In this way, a *laissez-faire* leader abdicates his/her responsibilities. Hence, a *laissez-faire* leadership style is not only a lack of presence, but a failure to meet the legitimate expectations of the subordinates and/or superiors concerned (Skogstad et al., 2017). In this way, there is an active element of withdrawal involved in this passive form of destructive leadership.

In the present study, we suggest that passive-avoidant leadership may negatively impact virtual team cooperation in several ways. For example, following Skogstad et al. (2007), avoidant leadership in terms of *laissez-faire* leadership may cause unclear expectations and role conflicts, as well as a diffusion of responsibilities. This may impair a teams’ ability to coordinate their efforts, and induce negative stress-reactions, that in turn impair work engagement and members’ willingness to contribute to the team (Song et al., 2019). It is likely that such lack of clarifications will have particularly negative effects in a virtual setting, given increased complexity related to team coordination – for example by difficulties in implicit coordination and the monitoring of other team members, which may hamper agile helping behavior as well as pushing (vs. pulling) of relevant information in the team (Salas et al., 2005; Brown et al., 2021). The relevance of such coordination in a virtual setting is supported by Johnsen et al. (2022), finding development of shared mental models, as a significant team coordination mechanism, to be the most frequent team behavior observed in virtual settings.

Moreover, collective norms are seen as an important antecedent of team cooperation (Taggar and Ellis, 2007; Song et al., 2019). If a leader fails in facilitating such norms, due to avoidance and lack of proactive control, the development of trust and cohesion may suffer. A state of uncertainty which also may hinder coordination of team behaviors, impair exchange of information, and cause conflicts within the team (Rusman et al., 2010). This is supported by Barling and Frone (2017), reporting an increase in frustration and conflict when leaders are perceived as *laissez-faire*. A leader that avoids the responsibility to actively engage and manage such conflicts may also facilitate a passive culture that accepts destructive behavior in a team, and as such cause a general breakdown of cohesion and subsequently cooperation (Dussault and Frenette, 2015).

Finally, following Skogstad et al. (2017), avoidant and *laissez-faire* leadership is related to an increase in workplace bullying and emotional fatigue, which in turn may impair team members ability and motivation to contribute to team processes. Ågotnes et al. (2018) found that a combination of co-worker conflicts and *laissez-faire* leadership increases the risk of team members defining themselves as victims of workplace bullying – which subsequently is likely to drain them of personal resources needed to contribute to the team. This may be particularly challenging in a virtual setting, where isolation and distancing between team members are high, possibly hindering social monitoring and a subsequent activation of social support and protection that may buffer the impact of such negative experiences. In the same vein, in a diary study across 36 days, Ågotnes et al. (2021) also found that daily *laissez-faire* leadership behavior increases the likeliness that daily work pressure enhances the respondents’ daily exposure to bullying-related negative acts among team members, which subsequently may obstruct team cooperation.

At this backdrop, we propose:

*Hypothesis 2*: Daily passive-avoidant leadership relates negatively to the quality of daily virtual team cooperation.

### The moderating role of task interdependence

Finally, we would argue that the impact of daily transformational and passive-avoidant leadership on virtual team cooperation is contingent of task interdependence in the team, referring to the extent each team member is dependent of assistance from the others, in terms of sharing of information, support, and materials, to be able to do their own job (Dyer et al., 2013; Brown et al., 2021). In other words, the extent to which interaction and coordination are needed to achieve the team objectives. Previous studies have shown that interdependent teams have members that interact and cooperate more frequently with each other (Potter and Balthazard, 2002), and are more effective, with higher levels of cohesion, compared to low interdependence (Langfred, 2005; Dyer et al., 2013; Arnold and Tafkov, 2019; Zhang et al., 2022). Kumar et al. (2009) also find that task interdependence facilitates performance and cooperation in virtual teams, possibly because such dependence requires frequent and effective sharing of information and decision making. Previous studies further report that members of task interdependent teams communicate and interact more frequently and with longer duration than others (Bjørn et al., 2014; Courtright et al., 2015). More so, Pinjani and Palvia (2013) find high interdependence positively related to trust between team members. Following De Jong et al. (2016), such trust is particularly important in virtual teams, given the limited opportunity for closer mutual control and regulation embedded in these remote teams.

Still, in line with Burke et al. (2006), we suggest that the increased frequency and quality of team cooperation needed in task interdependent teams may increase the importance of daily transformational leadership. Thus, as shown in previous diary studies, daily transformational leadership is stimulating important dynamic variables, like work engagement, self-strength use, and personal initiative (Breevaart et al., 2014; Bakker et al., 2022), which are closely related to individual performance and team orientation. Such daily initiative and engagement may be particularly important to circumvent contextual restraints of virtual teams (Wang and Howell, 2010). Conversely, the negative consequences of daily passive-avoidant leadership on daily team cooperation may be especially pronounced in teams of high task interdependency, given the need for daily coordination, information sharing, and mutual help and assistance, attributed to these teams (Burke et al., 2006; Bell et al., 2019). Hence, reactive and lack of leadership, for example in terms of impaired sharing of information and explicit coordination or avoidant approach to conflicts may here obstruct the individual team members’ ability to do their job properly, which in turn may evoke strong negative reactions and withdrawal from the team and the leader (De Jong et al., 2016). At this basis, we suggest:

*Hypothesis 3*: Task interdependency moderates the direct relationship between leadership and virtual team cooperation – so that (a) the positive effect of daily transformational leadership and (b) the negative effect of daily passive-avoidant leadership on the quality of daily virtual team cooperation is higher for teams of high task interdependency – compared to low.

## Method

### Design and procedure

The sample was based on data from the “Bergen home office study” – a five-day daily diary study with data collection starting in October 2020. Initially, three organizations were invited to participate with data collection (one shipping company, one public service organization, and one large hospital). They all allowed for us to recruit participants by an open invitation on their intranets. The volunteers picked up an unmarked envelope at their HR-department with an information letter, the questionnaires, and a return-envelope with our address. The data in the study was collected using a quantitative diary design (Ohly et al., 2010), where the respondents completed a standardized questionnaire at the end of every day for five consecutive working days. The daily questionnaire mapped fluctuating variables, which are assumed to vary from day to day, including the respondents’ evaluation of the quality of leadership and virtual team interaction. In addition to the daily questionnaire, the respondents were also asked to complete a general questionnaire which surveyed relatively stable individual variables, including the respondent’s assessment of their own task interdependence.

### Sample

The respondents were taken from a convenience sample of 214 employees from three organizations in Norway. The response rate was 28,5% on the general questionnaire, yielding 61 person-level observations at Level 2. Of these, 58 employees completed the daily questionnaires, yielding 290 day-level observations on Level 1 (out of 305 possible day-level observations). Accordingly, the response rate on the daily questionnaire was 95%. The final sample consisted of 39 women (67,2%) and 19 men (32,7%). The mean age of the respondents was 47 years, with a range from 23 to 64 years (SD = 10.4). In terms of professional roles, all participants were “office workers” in either HR, finance, or logistics departments. Three respondents did not report their age.

### Ethics and data processing

To ensure good data processing and ethical considerations, the study was registered in the System for Risk and compliance (RETTE) at the University of Bergen, prior to initiation of the project. All respondents were presented with an informed consent form, in which they were also informed of the possibility to withdraw from the study at any time. They were also informed that all data would be treated confidentially.

### Instruments

All study variables were measured using quantitative daily diaries, with adapted versions of existing scales. The time frame of the scales and the number of questions were adapted so the questions could be answered on a daily basis (*cf.* Ohly et al., 2010). Reliability of the measures was calculated using the approach described by Geldhof et al. (2014) by estimating omega (*ω*) at the between-person level, as well as the within-person level for the daily measures, using a two-level CFA. The daily questionnaire was reviewed and tested to avoid dropout and exhaustion related to repeated responses (Ohly et al., 2010). The average response time on the questionnaire was between 5 and 7 min, which is in line with Reis and Gable’s (2000) recommendations to reduce possible exhaustion and dropout.

#### Team task interdependency

Team task interdependency is a person-level variable, measured using the subscale for ‘task interdependence’ by Van der Vegt et al. (2001). This scale consists of five items, with response categories ranging from 1 (*strongly disagree*) to 5 (*strongly agree*). Examples of items from the scale are “I depend on my colleagues for the completion of my work” and “I have to work closely with my colleagues to do my work properly.” The scale showed an internal consistency (ω) of 0.83 at the between-person level.

#### Day-level virtual team cooperation

Daily virtual team cooperation was measured using six of the original 17 items from the Team Interaction Scale (Song et al., 2019). The original scale was shortened to fit better with the daily diary design. This selection was performed by retaining the item with the highest factor loading in the original publication (Song et al., 2019) for each of the six factors in team interaction. In the present sample, the standardized factor loadings for the six included items ranged from 0.63 to 0.75. The scale was scored using a 7-point Likert scale, ranging from 1 (*completely disagree*) to 7 (*completely agree*). For example, the factors communication and conflict management were measured by the following items, respectively: “Team members communicate intensively with each other,” and “disagreements between the team members are solved rapidly.” The scale showed an internal consistency (ω) of 0.96 at the between-person level and 0.79 at the within-person level. This indicates that the chosen items included in the present study represent a reliable measurement of virtual team interaction.

#### Day-level transformational leadership

Daily transformational leadership was measured using the Global Transformational Leadership scale (GTL) developed by Carless et al. (2000). The scale consists of seven items and was answered on a 5-point Likert scale, rated from 1 (*completely disagree*) to 5 (*completely agree*). Examples of items are: “During the working day, my immediate leader has”: “… communicated a clear and positive vision of the future,” and “… fostered trust, involvement and cooperation among team members.” The scale showed an internal consistency (ω) of 0.85. at the between-person level and 0.98. at the within-person level.

#### Day-level passive-avoidant leadership

Daily passive-avoidant leadership was measured using the Passive Leadership scale by Barling and Frone (2017), which includes two items adapted from Pearce and Sims (2002), and two items adapted from Den Hartog et al. (1997). This five-item scale consists of two items measuring management-by-exception-passive and three items measuring *laissez-faire* leadership. The items were answered using a 5-point Likert scale, ranked from 1 (*completely disagree*) to 5 (*completely agree*). Examples of items are: “During the working day, my immediate leader has”: “… avoided making decisions” and “waited for things to go wrong before taking action.” However, two-level CFA revealed a low factor loading (0.23) for one of the items (“During the working day, my immediate leader has been unavailable when staff needed help with a problem”). This item was therefore excluded, resulting in an internal consistency (ω) of 99. at the between-person level and 0.85. at the within-person level for the four remaining items.

### Strategy of analysis

We estimated multi-level correlations and reliability using Mplus 7.4 (Muthén and Muthén, 2010). In order to capture the multilevel structure of the data, which implied that the five daily measurements (level 1) of the study constructs were nested within individuals (level 2), hypothesis testing was carried out with multilevel analyses using MLwiN 3.01 (Rasbash et al., 2017). In the analyses, the level 1 (day-level) predictors were centered on the respective person’s mean, while the level 2 (person-level) variable was centered on the grand mean.

First, we tested an unpredicted model (null model) to investigate how much of the variance in the dependent variable (virtual team cooperation) that could be explained by variations within the individual (day-level) and how much could be explained by variations between individuals (person-level). Subsequently, a main effects model was tested to examine the direct relationship between the independent variables (transformational leadership and passive-avoidant leadership) and the dependent variable. Finally, we estimated an interaction effects model to analyze whether the level of task interdependence moderates the relationship between transformational leadership and passive-avoidant leadership, and virtual team cooperation, respectively.

## Results

### Descriptive statistics

Means, standard deviations, and day- and person-level correlations for all study variables are presented in Table 1.

**Table 1:** Mean, standard deviation, and correlations for the study variables.

	Scale	M	SD	ICC^a^	1	2	3	4
Day-level								
1. Virtual team cooperation	1–7	4.86	1.36	0.722	-	0.21**	0.10	–
2. Transformational leadership	1–5	3.27	0.69	0.678	0.67**	–	−0.38**	–
3. Passive-avoidant leadership	1–5	2.04	0.74	0.641	−0.51**	−0.62**	–	–
Person-level								
4. Task interdependency	1–5	3.59	0.66	–	0.31**	0.16	−0.20	–

^a^ICC = Person-level intraclass correlation. Correlations above the diagonal are correlations on the within (day) level and correlations below the diagonal are correlations on the between (person) level. ∗∗*p* < 0.01, ∗*p* < 0.05, *N* = 61 respondents, N = 305 observations.

As shown in Table 1, significant correlations at the within-person level are found between transformational leadership and virtual team cooperation (*r* = 0.21, *p* < 0.01), and passive-avoidant leadership (*r* = −0.38, *p* < 0.01), respectively. However, no significant correlation is found between passive-avoidant leadership and virtual team cooperation.

At the between-person level, there is a significant negative correlation between transformational leadership and passive-avoidant leadership (*r* = −0.62, *p* < 0.01), and a significant positive correlation between transformational leadership and virtual team cooperation (*r* = 0.68, *p* < 0.01). Furthermore, there is a negative correlation between passive-avoidant leadership and virtual team cooperation (*r* = −0.51, *p* < 0.01). Finally, the results show a significant positive correlation between virtual team cooperation and task interdependence (*r* = 0.31, *p* < 0.01).

### Multi-level analysis

The results from the multi-level analysis of the prediction of daily virtual team cooperation are presented in Table 2. First, we tested an unpredicted (null-model) to confirm that there is sufficient day-level (within subjects) variance in the dependent variable. As shown in Table 2, the initial unpredicted model revealed significant variation in experienced virtual team cooperation at both the day-level (28%) and the person-level (72%).

**Table 2:** Multilevel estimates for the prediction of daily virtual team cooperation.

	Null model		Main effects model		Interaction model	
	B	*SE*	B	*SE*	B	*SE*
Intercept	4.867**	0.134	4.894**	0.128	4.894**	0.128
Transformational leadership			0.267*	0.090	0.234*	0.092
Passive-avoidant leadership			−0.037	0.083	−0.033	0.083
Task interdependence			0.410**	0.151	0.410**	0.151
Interaction 1^a^					0.179	0.100
Interaction 2^b^					0.078	0.097
Variance level 1 (day-level)	0.372 (28%)	0.035	0.353	0.034	0.348	0.033
Variance level 2 (person-level)	0.975 (72%)	0.194	0.873	0.176	0.874	0.176
*−2 Log Likelihood*	678.70		643.05		639.77	

^a^Interaction 1 = Transformational leadership*task interdependence, ^b^Interaction 2 = Passive-avoidant leadership*task interdependence. *p < 0.05, **p < 0.01, *N* = 58 respondents, *N* = 276 observations

In support of hypothesis 1, the results from the main effects model showed a significant effect of daily transformational leadership on daily virtual team cooperation (B = 0.267, *p* < 0.05). We did not find support for hypothesis 2, in which we expected daily passive-avoidant leadership to have a negative effect on daily virtual team cooperation (B = − 0.037, *n.s.*). Furthermore, the results showed that the person-level variable task interdependence was positively related to daily virtual team cooperation (B = 0.410, *p* < 0.01). However, contrary to our predictions in hypothesis 3, the interaction model showed no significant interaction between task interdependence and neither transformational- (B = 0.179, *n.s.*) nor passive-avoidant leadership (B = 0.078, *n.s.*) on daily virtual team cooperation.

## Discussion

The Covid-19 pandemic led to a worldwide transformation of work-organization, from traditional office-settings to home-office arrangements and virtual team cooperation. In the literature, a growing body of studies provides insights on how to manage such virtual cooperation (e.g., Garro-Abarca et al., 2020; Kashive et al., 2022; Kilcullen et al., 2022). Few, however, have studied the link between effective virtual cooperation and leadership – and few if any have investigated how dynamic processes that may change rapidly, independent of stable trait-like differences, may explain this relationship. The current study shows that about one third of the variance in virtual team cooperation can be attributed to such day-to-day dynamic processes across five consecutive days, which in turn suggests that such cooperation is highly malleable and in need of daily follow up by a leader in line with functional leadership theory (Brown et al., 2021). We further found that at days when leaders show transformational leadership behaviors, the cooperation in the virtual teams is perceived as better. On the other hand, unexpectedly, we found daily passive-avoidant leadership unrelated to the daily team cooperation. Furthermore, even though task interdependence did have a direct effect on virtual team cooperation, it did not moderate the relationship between daily leadership and team cooperation. Below we discuss the main theoretical contributions in more detail.

### Theoretical contributions

The present study makes several contributions to the literature. First, in line with hypothesis 1, our study lends support to recent studies finding transformational leadership a particularly relevant approach to the management of virtual teams. Our findings are in line with Sedrine et al. (2021), who show a positive relationship between transformational leadership and both performance and satisfaction in virtual teams, mediated through trust and operational cohesion. As such, transformational leadership represents influence-strategies of long-range impact – transcending physical contact, as suggested in early developments of this theory (Bass, 1985). Following functional leadership theory, which portray the role of leadership as mainly to attend to whatever is not being dealt with to satisfy the needs of the team (Brown et al., 2021), this suggests that daily transformational leadership addresses the needs of virtual teams in an effective way. As mentioned in the introduction, this may for example be the provision of a shared direction and subsequent coordination, as well as the daily encouragement of each team member to participate actively in the team leadership processes, which in turn strengthens the collective leadership process. Furthermore, in line with Palos-Sanchez et al. (2022), a transition from face-to-face teamwork into a virtual setting, may cause impairment in cohesion, and a weakened sense of belonging between the team members, due to issues like more frequent impersonal social exchanges and reduced contact points between the team-members. Accordingly, the inclusion of avatar technology (e.g., “Gather town” virtual spaces) may reduce this risk. However, our study suggest that also transformational leadership may buffer such a decline in cohesion, by stimulating a common purpose, and instilling a collective mindset among the team members (e.g., Bass and Riggio, 2006) – which in turn stimulate the quality of daily teamwork. It is also worth mentioning that transformational leadership may encompass both a relationship- and a task-oriented leadership approach, as well as charismatic influence, by focusing on common goals, idealized role modeling and the supporting and development of each individual team member (Bass and Riggio, 2006). As such, this may be a more effective and comprehensive approach, compared to for example the relationship – vs. task orientation taxonomy utilized by Brown et al. (2021). Second, our study supports the notion that transformational leadership has a particularly relevant role in times of crisis and difficult situations (Bass and Riggio, 2006). The study was conducted during the first phase of Covid-19, a period with high levels of uncertainty, anxiety, and frustration in the society regarding the consequences and development of the pandemic – combined with government enforced transition from office to virtual work for many. Our study shows that by daily provision of positive interpretations of the situation, meaning and hope, along with stimulation of creative thinking and individualized consideration – the best leaders were able to knit people together and enhance daily team cooperation, even during virtual conditions. This aligns with Weinberg’s (1978) review of 30 cases of team responses to nature-disasters like earthquakes and hurricanes, finding provision of vision and individualized support especially relevant for team performance. Third, and contrary to our expectations in hypothesis 2, the present study shows that negative effects of passive-avoidant leadership found in previous studies of work with physical presence (e.g., Skogstad et al., 2014), did not emerge in the virtual setting. This is surprising, given that positive outcomes attributed to transformational leadership in previous studies (Judge and Piccolo, 2004), and the well documented negative outcomes connected to *laissez-faire* leadership in face-to-face settings (e.g., Skogstad et al., 2014). More so, several studies have shown that passive and avoidant leadership is related to a variety of negative outcomes, including fatigue, frustration, and reduced initiative at work, which could be expected to impair team cooperation (Skogstad et al., 2014). However, we could speculate that this unexpected finding is the result of changes in followers’ expectations and demands related to their leader, and what a leader should contribute during challenging virtual condition like this. It is possible that followers recognize and accept the difficulties leaders are facing during such difficult times, including virtual conditions. As such, their reactions to reduced leader presence and availability, as well as less proactive monitoring and agile responding, may be more accepting, and less negative, compared to “normal” office settings. Such acceptance may also lead to an increased acceptance of personal responsibility for the work-situation and the team processes, which may increase the level of self-management and leadership substitutes - compensating for the avoidance of the leader (e.g., Kerr and Jermier, 1978). However, it is possible that more active forms of destructive leadership, like abusive supervision or ostracism may be more relevant perspectives in virtual teamwork (e.g., Skogstad et al., 2017). For example, it may be easier for a leader to exercise “silent treatment” strategies from a distance, combined with less possibility for the rest of the team to compensate for such “freeze out” tactics, when team members are physically dispersed. These things may go “under the radar” for the rest of the team and, as such, be more hurtful compared to a physical setting, where social support may buffer the detrimental effects of such leader behavior. More studies of other forms of destructive leadership on virtual team cooperation is thus warranted.

Fourth, our study shows that task interdependence of a team has no impact on the relationship between daily leadership and virtual team cooperation – as perceived by the individual team members. As outlined in hypothesis 3, this is surprising, given previous studies showing that task-interdependent teams interact and cooperate more frequently with each other (Potter and Balthazard, 2002) – which could suggest that these teams have a greater need for leader-coordination and stimulation of team cooperation, compared to other teams. This also contrasts Brown et al.’ (2021) finding of task interdependence as a moderator of both relation- and task-oriented leadership, which may overlap with transformational leadership. Given our finding of a positive relationship between task interdependency and daily team cooperation, but no moderation effect, one explanation may be that this form of task interdependency has served as a source of leadership substitute, seen as social processes that reduces the relevance of formal leadership influence (Kerr and Jermier, 1978). Through frequent daily virtual interactions between the team members, without the presence of the leader, it is likely that collective leadership processes and the ability to self-manage is developing, thus rendering the daily contribution from the leader less relevant – and possibly at the level of less inter-dependent teams. It may also be that the elevated level of cohesion found in highly task-interdependent virtual teams, may have reduced the importance of leadership influence as such in these contexts (Zhang et al., 2022).

Finally, a methodological contribution is our use of a daily quantitative diary design following remote workers at their home office for 5 consecutive days. Although several studies have now used a within-person perspective to study leadership, most studies have used a between-person design to uncover how leadership traits or styles are linked to outcomes like individual work performance (Bakker et al., 2022). Notably, few have utilized a quantitative diary design in team-research. Most of this research utilizes between-teams designs (e.g., Johnsen et al., 2022) – with the underlying assumption that team behavior stems from stable team characteristics. An example is team-role theories, like Belbin (2010), linking personality-based team roles, and the distribution of these, to effective team cooperation and performance. An alternative approach is focus on team behavior (e.g., Salas et al., 2005). Given previous studies showing significant within-subject variation from day to day of a multitude of work-related variables in leaders and followers (e.g., Ohly et al., 2010), we suggest similar processes influencing team- cooperation. Hence, the quality of team cooperation may fluctuate to some degree from day to day - caused by variables that are changeable over a relatively short time span, like emotions or social perceptions (e.g., Ohly et al., 2010). In other words, there may be psychological processes within each individual crew member, or team, that may be sensitive to situational influences, and that fluctuates in patterns partially unrelated to dispositions. A challenge in the further development of this methodology to team-research, however, is the risk of cross-categorization between levels of analysis. To circumvent this problem in the current study, we measured team cooperation as individual team-members’ evaluation of team performance from day to day. This may, however, be a rather inaccurate representation of actual team cooperation, better measured by use of external expert ratings of team behavior. More work on how to utilize this methodology on team-level research is warranted, to better capture the dynamic aspects of team cooperation, and thus, provide more nuanced theories, combining stable and dynamic processes as predictors and mechanisms.

### Limitations

The present study makes several contributions but is not without limitations. First, the predictors and outcome variable are measured by the same respondent, increasing the risk of common methods biases (Podsakoff et al., 2003). To avoid this, researchers could utilize expert ratings or peer ratings (of daily individual team behavior). In the same vein, even with several consecutive days of measuring the relationship between leadership behavior and team cooperation, the predictor and outcome variables were assessed at the same moment each day, which makes it impossible to determine causality. In future research this can be circumvented by including lagged effects in the model, for instance testing the effect of daily leadership on team cooperation the following day (e.g., Bakker et al., 2022). Furthermore, the measure of team cooperation is more specifically the individual team members’ daily evaluation of team cooperation. Such evaluations may also be influenced by shifts in emotions or experiences of the evaluator, which in turn may reduce the accuracy of the assessment, and possibly reduce the validity of the measure (Podsakoff et al., 2003). One way of tackling this may be to adjust the team interaction scale from team behavior into individual team behavior and utilize second source ratings of this behavior. Ideally, the study should also include more days, a longer time span, to better capture the dynamic influences on daily team cooperation. However, due to the strain the respondents worked under during the pandemic, we chose to limit the burden on the respondents as much as we could. Furthermore, an interesting variation to the convenience sampling of the current study, could be to pick specific teams with team-members that work together daily. This would enable second source ratings of performance and behavior, reducing the risk of common source biases, and could also open for investigations of team characteristics as moderators – measured on team-level. Another limitation worth mentioning is that transformational leadership was measured on a short scale that treats the concept as an overall construct – to avoid overburdening the participants with lengthy questionnaires. However, the consequence was that we were unable to investigate the relationships of the separate facets of transformational leadership with team cooperation. Future diary research could benefit from studying the unique contribution of idealized influence, inspirational motivation, intellectual stimulation, and individual consideration – to better capture the nuances of transformational leadership influence on team cooperation. Further, a potential limitation is related the fact that while we include the team members task interdependence as a contextual factor in our analyses, we do not control for the degree to which individuals’ rewards are based on their performance as a group (outcome dependence; Van der Vegt et al., 2001). However, it is possible that task interdependence is only related to virtual team co-operation in situations when the team members are also dependent on each other to achieve a reward. Thus, future studies should take this into consideration. Future studies could also control the effect of possible interpersonal differences in available computer and video technology, as well as cultural diversity, on the relationship between virtual leadership and team cooperation (e.g., Marino-Romero et al., 2022).

### Practical implications

The present study has several implications for human resource practices aimed at enhancing virtual team cooperation in organizations. First, the results emphasize the relevance of transformational leadership as a goal for leadership training also of virtual team leaders, as well as the coaching and supervision of these leaders. This may be particularly true in highly complex and uncertain work contexts, as experienced during the Covid 19 pandemic. In such a context, lack of vision and creative thinking, as well as inspirational motivation and individualized consideration may severely impair the dynamics of the virtual team. Second, the finding that passive-avoidant leadership was unrelated to daily virtual team cooperation, may suggest that organizations should focus on the constructive sides of leadership in leadership assessments and evaluations, and that virtual leaders should be selected at the basis of their ability to develop and inspire, more than their ability to be present and available. Hence, in a virtual setting, good seems stronger than bad, at least in terms of stimulation of team cooperation. Third, given the findings that a significant portion of the quality of virtual team cooperation is driven by malleable and dynamic processes, unrelated to stable traits, team leaders need to be attentive and enact transformational leadership strategies daily to maintain a high level of team cooperation, and reduce number of bad days for the team. Thus, leaders should strategically use transformational leadership behaviors, such as idealized influence or individual consideration, contingent on the daily state of the team. Finally, task interdependence seems to work as a leadership substitute in this study. This suggests that teams of lower task interdependence may need a closer follow up, to facilitate team cooperation not driven by highly interdependent work processes in the team.

## Conclusion

The current study finds that a substantial portion of the variance in virtual team cooperation can be attributed to day-to-day dynamic processes within the team, which indicates that team cooperation is a malleable process, and thus a process that should be nurtured daily. The study shows as expected that the daily enactment of transformational leadership behaviors from a virtual leader is important for the quality of daily virtual teamwork. When leaders are idealized, show individual consideration, and/or are motivating and intellectually stimulating, they can enhance features like effective communication and agile coordination, as well as shared team goals, helping behavior and conflict resolution in virtual teams. Unexpectedly, the study further find that a passive and avoidant leader has little or no impact on the daily virtual team cooperation – which in sum suggests that in virtual settings inspirational and development- oriented leadership is what the teams need – while they are less vulnerable to lack of leadership. Hence, in virtual settings, “good is stronger than bad” in terms of the relative negative effect of destructive leadership compared to the positive effect of constructive and inspirational leadership. We hope that our study will provide a source of inspiration for leadership scholars to validate and expand the proposed model, by including mediators and other moderators, as well as team level assessments of performance.

## Data availability statement

The raw data supporting the conclusions of this article will be made available by the authors, without undue reservation.

## Ethics statement

The studies involving human participants were reviewed and approved by RETTE University of Bergen. The participants provided their written informed consent to participate in this study.

## Author contributions

OO: developed the project, research model, preparing and recruiting participants, developing hypothesis, and analysis and discussion. KÅ: developing research model, collecting data, participating in theory development, analysis and results, and discussion. JH: development of research model and analysis strategies, theory development, and analysis and discussion. RE: participated in data collection, theory development, and analysis and discussions. CR: recruitment of participants, theory development, and analysis and discussion. All authors contributed to the article and approved the submitted version.

## Conflict of interest

The authors declare that the research was conducted in the absence of any commercial or financial relationships that could be construed as a potential conflict of interest.

## Publisher’s note

All claims expressed in this article are solely those of the authors and do not necessarily represent those of their affiliated organizations, or those of the publisher, the editors and the reviewers. Any product that may be evaluated in this article, or claim that may be made by its manufacturer, is not guaranteed or endorsed by the publisher.
